# Sonographic Detection of Iatrogenic Carotid Artery Guidewires During Internal Jugular Vein Catheterization 

**DOI:** 10.24908/pocus.v9i2.17366

**Published:** 2024-11-15

**Authors:** James H Moak, Kristen C Swann, Matthew M Kongkatong, Jakob E Ottenhoff, Christopher D Thom

**Affiliations:** 1 Department of Emergency Medicine, University of Virginia Health System Charlottesville, VA USA; 2 Department of Emergency Medicine, Kaiser Permanente San Rafael Medical Center San Rafael, CA USA

**Keywords:** Ultrasound, Internal Jugular Vein, Carotid Artery, Catheterization, Guidewire, Iatrogenic

## Abstract

**Background:** Visualization of the guidewire during internal jugular (IJ) vein catheterization by point of care ultrasound (POCUS) has been recommended for avoiding inadvertent carotid artery dilation. The purpose of this study was to determine the accuracy of POCUS for identifying guidewires inappropriately placed in the carotid artery. **Methods:** This prospective, observational study involved emergency medicine (EM) residents with varying experience in guidewire visualization. Using an inanimate model, investigators placed guidewires randomly into the carotid artery or IJ vein. Residents, blinded to guidewire location, scanned the model and recorded their findings. The test performance of POCUS for arterially placed guidewires was evaluated through calculation of sensitivity, specificity, negative predictive value (NPV), positive predictive value (PPV), and overall accuracy, using investigator placement as the non-reference standard. **Results: **Twenty-five residents performed 51 observations. The test performance of POCUS for identifying arterially placed guidewires was sensitivity 95.0% (95%CI = 73.1-99.7%), specificity 96.8% (95%CI = 81.5-99.8%), NPV 96.8% (95%CI = 81.5-99.8%), and PPV 95.0% (95%CI = 73.1-99.7%). The overall accuracy was 96.1% (95%CI = 86.8-98.9%). Residents reported being very confident in their findings in 88.2% of all observations (95%CI = 76.6-94.5%), somewhat confident in 9.8% (95%CI = 4.3-21.0%), and not very confident in 2.0% (95%CI = 0.4-10.3%). No errors occurred among upper-level residents (post-graduate years 2-3) or those reporting >5 prior wire visualizations in live patients. **Conclusions:** This study is the first to demonstrate that physicians can easily identify misplaced guidewires located in the carotid artery with a high degree of accuracy using POCUS. We recommend routine scanning of the IJ vein and carotid artery prior to vessel dilation to reduce the likelihood of carotid artery injury.

## Background

Visualization of the guidewire during internal jugular (IJ) vein catheterization using point of care ultrasound (POCUS) has been proposed as a means of avoiding iatrogenic carotid artery cannulation 1, 2, 3, 4, 5. Prior studies examining the accuracy of this technique have not involved arterially placed guidewires. The purpose of this study was to determine the accuracy of POCUS for identifying guidewires placed in the carotid artery rather than the IJ vein. We hypothesized that physicians in training with varying experience levels would have a high degree of accuracy identifying a misplaced guidewire in the carotid artery of an inanimate vascular access model. 

## Methods

This was a prospective observational study involving post-graduate year (PGY) 1-3 emergency medicine residents at the University of Virginia. The study took place on nine different occasions over a seven-month period alongside regularly scheduled resident didactics and involved a convenience sample of volunteers in attendance. The University of Virginia Institutional Review Board for Health Sciences Research approved the study protocol. All participants provided written informed consent. 

The study relied upon a single inanimate vascular access model (CAE Blue Phantom, Sarasota, FL) equipped with a simulated IJ vein and carotid artery amenable to POCUS visualization. The carotid artery was connected to a pneumatic bulb, which could be squeezed in a rhythmic fashion to generate pulsations. Participants used a Sonosite M-Turbo ultrasound machine (Bothell, WA) with a 6-13 MHz, 25 mm linear array transducer to scan the model. 

In preparation for each study observation, one of the investigators used ultrasound guidance to place a guidewire percutaneously into the carotid artery or the IJ vein based on simple randomization (i.e., coin toss). A second investigator scanned the wire to verify agreement with its intended location prior to testing. Only one resident was allowed in the testing area at a time. Participants, blinded to guidewire location, scanned the simulator using the linear array transducer, while an investigator generated carotid pulsations with the pneumatic bulb that were commensurate with a normal heart rate. Whether to scan the wire transversely, longitudinally, or both was left to the discretion of each participant. Participants were not allowed to manipulate the guidewire during imaging. After scanning the model, participants recorded the location of the wire on a data sheet. Subjects also recorded their level of training, prior experience visualizing guidewires (number of experiences: None, <5, 6-10, 11-15, 16-20, 21-25, or >25), and degree of confidence in their findings (not very confident, somewhat confident, or very confident). For convenience of enrollment, residents could participate in the study more than once, but no more than once on the same day. There was no dedicated teaching or training session prior to participants’ enrollment in the study, though guidewire visualization is encouraged in our department as a routine step in central line placement. 

Five measures of test performance were calculated to evaluate the utility of POCUS for identifying arterially placed guidewires: sensitivity, specificity, negative predictive value (NPV), positive predictive value (PPV), and overall accuracy. Investigator-confirmed placement was used as the non-reference standard. Additionally, we evaluated residents’ degree of confidence in their POCUS findings. Errors in the identification of guidewire placement by ultrasound were compared across participant characteristics, including level of training, prior experience, perceived confidence in sonographic findings, and number of times participating in study. We report proportions with a 95% confidence interval (CI) where appropriate.

**Table 1:** Test characteristics for visualization of the guidewire in the carotid artery or internal jugular vein in an inanimate vascular access model using POCUS.

	Guidewire placed in artery	Guidewire placed in vein
Guidewire observed in artery by POCUS	19	1
Guidewire observed in vein by POCUS	1	30

Sensitivity for misplaced (arterial) wires was 95.0% (95%CI = 73.1-99.7%), specificity 96.8% (95%CI = 81.5-99.8%), negative predictive value (NPV) 96.8% (95%CI = 81.5-99.8%), and positive predictive value (PPV) 95.0% (95%CI = 73.1-99.7%). The overall accuracy was 96.1% (95%CI = 86.8-98.9%).

## Results

Twenty-five residents (seven PGY-1s, nine PGY-2s, and nine PGY-3s) participated in the study. Two had no prior experience in guidewire visualization in a live patient; nine reported <5 prior attempts, five reported 6-10, three 11-15, two 16-20, and three 21-25. A total of 51 wire observations occurred in the study. Nine residents participated only once, nine of them participated twice, five of them three times, one four times, and one five times. Randomization resulted in 20 wires placed in the carotid artery, and 31 in the IJ vein (See Table 1). The test performance of POCUS for identifying wires in the carotid artery was sensitivity 95.0% (95%CI = 73.1-99.7%), specificity 96.8% (95%CI = 81.5-99.8%), NPV 96.8% (95%CI = 81.5-99.8%), and PPV 95.0% (95%CI = 73.1-99.7%). The overall accuracy was 96.1% (95%CI = 86.8-98.9%). Residents reported being very confident in their findings in 88.2% of all observations (95%CI = 76.6-94.5%), somewhat confident in 9.8% (95%CI = 4.3-21.0%), and not very confident in 2.0% (95%CI = 0.4-10.3%) (See Table 2). No errors occurred among upper-level residents (PGY 2-3), residents reporting >5 prior wire visualizations in live patients, or residents who reported being very confident in their findings. One error occurred during a participant’s first and only time participating in the study; another occurred during a participant’s second of two observations. 

Excluding the two operators who participated more than three times from the data set yielded the following test characteristics: sensitivity 94.4% (95%CI = 70.6-99.7%), specificity 95.8% (95%CI = 76.9-99.8%), NPV 95.8% (95%CI = 76.9-99.8%), PPV 94.4% (95%CI = 70.6-99.7%), and overall accuracy 95.2% (95%CI = 82.6-98.6%). Excluding all attempts after each participant’s first attempt resulted in the following test performance: sensitivity 100.0% (95%CI = 67.9.-100%), specificity 92.9% (95%CI = 64.2-99.6%), NPV 100% (95%CI = 71.7-100%), PPV 91.7% (95%CI = 59.8-99.6%), and overall accuracy 96.0% (95%CI = 80.5-99.3%).

**Table 2:** Confidence of participants in their assessment of guidewire location.

Actual guidewire location	Level of Confidence		
	Not very confident	Somewhat confident	Very confident
Carotid artery	0 correct 0 incorrect	2 correct 1 incorrect	17 correct 0 incorrect
Internal jugular (IJ) vein	1 correct 0 incorrect	1 correct 1 incorrect	28 correct 0 incorrect

## Discussion

Iatrogenic puncture, or worse, dilation of the carotid artery is a potentially devastating complication of central venous catheterization that can lead to severe bleeding, hemothorax, carotid-jugular fistula, stroke, and death 6, 7, 8. The use of real-time ultrasound guidance during IJ vein cannulation significantly decreases the likelihood of carotid injury but does not completely eliminate this risk 9, 10, 11. Visualization of the guidewire before dilation and catheter placement using POCUS has been proposed as a means of lowering the risk of inadvertent carotid artery cannulation 1, 2, 3. This step has been incorporated into routine practice guidelines for IJ vein catheterization by several specialty organizations and institutions 12, 13, 14, 15, 16. The guidewire appears to be easy to visualize, with rates reported as high as 89% for trainees^2^ and 100% for experts^3^ during successful IJ vein catheterization in actual patients. Trainees are also able to identify the location of guidewires with 97% accuracy as they course through a target vessel, or run errantly beside it, in an inanimate gelatin model 1. 

**Figure 1:**
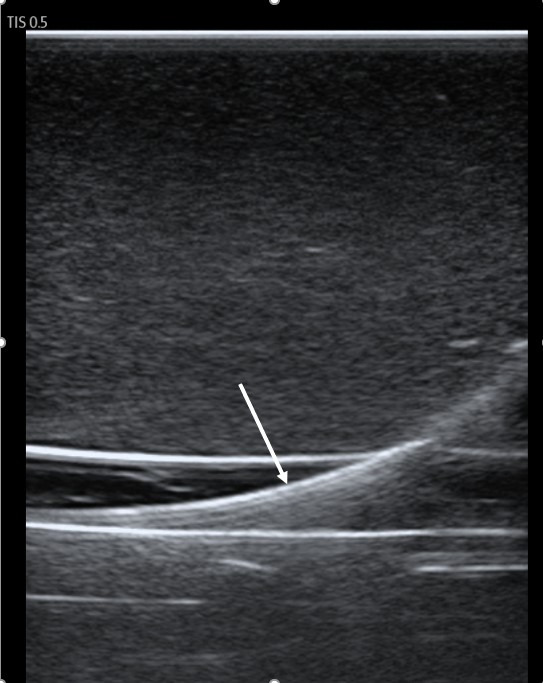
Longitudinal (in-plane) view of the guidewire (arrow) coursing through the carotid artery in an inanimate vascular access model.

**Figure 2:**
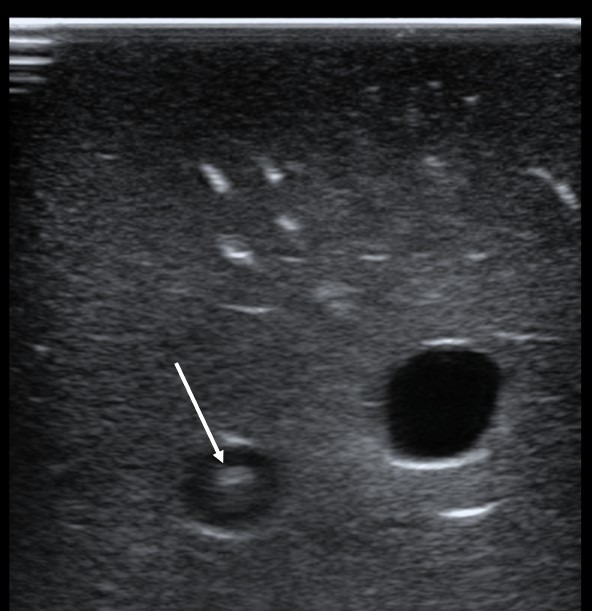
Transverse (out-of-plane) view of the guidewire (arrow) coursing through the carotid artery in an inanimate vascular access model. Note that no guidewire is demonstrated in the internal jugular (IJ) vein at right.

Since intentional cannulation of the carotid artery in an actual patient would be unethical, a paucity of research exists on the accuracy of sonographic identification of guidewires inadvertently placed in the carotid artery. This study appears to be the first to examine this question, by using an inanimate vascular access model with a pulsatile carotid artery. Our data suggest that, at least under simulated circumstances, physicians with varying degrees of experience can distinguish between guidewires located in the carotid artery versus the IJ vein with a high degree of accuracy. Among all visualization attempts, our study only yielded one false negative (wire mistakenly identified as in the IJ vein) and one false positive (wire mistakenly identified as in the carotid artery). Both errors were committed by first year residents with fewer than five prior attempts at guidewire identification. Of note, in both instances the operators reported being only “somewhat confident” in their findings. 

The optimal number of training scans needed to approach perfection at identifying arterially placed guidewires is unknown. Our data suggest that at least five training scans are needed, though the true minimum required for competency remains difficult to gauge. Based on our experience we suspect this number to be less than twenty-five, a figure cited by expert operators in a previous study 3. Since proficiency in one ultrasound-guided procedure, such as nerve blockade or foreign body removal, likely enhances skills in another, a uniform, numerical benchmark for readiness in guidewire visualization may prove elusive. 

As a safety measure for IJ vein cannulation, guidewire visualization in the proper target vessel prior to dilation seems intuitively beneficial. This technique, however, may not be foolproof. At least one author has reported carotid artery cannulation despite prior wire confirmation in the IJ vein 17. The needle, and subsequently, the wire was thought to have traversed the lumen of the vein en route to a more posteriorly located artery. Others have reported inadvertent cannulation of the subclavian artery after guidewire visualization in the IJ vein 18, 19. It appears that how the vessels and wire are scanned is relevant. In one of the above cited cases of subclavian artery cannulation, the operator was unable to obtain a longitudinal (in-plane) view of the wire due to its proximity of the clavicle 18. We concur with other authors that a longitudinal view of the guidewire is important to obtain 20. Such a view permits the operator to observe the course of the wire over several centimeters as it passes into the lumen of the vessel and continues distally. Figure 1 demonstrates this longitudinal approach with the guidewire placed in the carotid artery of the vascular access model used in our study. Figure 2 shows the wire in the same vessel in a transverse (out-of-plane) view. When using the latter approach, the wire should be visualized within the IJ vein and followed along its course to ensure that it does not proceed distally through the posterior wall of the target vessel and into the carotid artery. In our experience, obtaining both longitudinal and transverse views takes little additional time and may confer additional certainty of the wire’s position. 

Our results must be interpreted in light of several limitations. Guidewire visualization in the carotid artery may be more challenging in a live patient than in an inanimate model. Still, we have no reason to suspect that to be the case. Human studies examining venous guidewire identification in adults^2^, ^3^, ^4^, ^5^ and children^20^ reveal high success rates comparable to those of our investigation and to those of a previous study involving an inanimate vascular access model 1. The pulsatility of the carotid artery may, in fact, make visualization of a guidewire within this vessel easier and even more striking than when located within the IJ vein. Nonetheless, we recognize that body habitus is a factor in successful guidewire visualization 5, 20. Our vascular access model simulates a non-obese, adult patient who may be easier to scan than some patients requiring central venous catheterization. Hence, our findings may not be generalizable to actual patients undergoing IJ vein cannulation. Animal or cadaveric studies may provide additional inferences on the ease of arterial guidewire identification in live human subjects. Also, our vascular access model does not simulate the presence of a posteriorly located carotid artery that could be mistakenly penetrated by a needle, then a guidewire, after initially traversing the IJ vein. Hence, our study provides no insight on how challenging it might be for an operator to identify this type of errantly placed guidewire. We suspect, however, that a single longitudinal view of the wire would alert the operator to this possibility. 

An additional limitation of our study is that we did not seek to measure the time required for operators to identify the location of the guidewire. Like other investigators, we believe this step in IJ vein cannulation takes little additional time 1, 2, 3, 4. We are unable to comment on why the two errors in our study occurred beyond attributing them to operator inexperience. Had we captured data on time required to scan the model or which views the operator undertook, we may have gained more insight on why these errors occur. Finally, the design of our study did not allow for participants to manipulate the guidewire manually during scanning. This step, recommended by other authors when encountering difficulty 12, could have led to even fewer number of errors than observed in our study. 

## Conclusions

Identification of the guidewire by POCUS prior to dilation of the vessel has been increasingly recognized as an important precautionary step during IJ vein catheterization. This study, conducted on an inanimate vascular access model, is the first to demonstrate that operators can easily identify misplaced guidewires located in the carotid artery with a high degree of accuracy. We encourage routine scanning of the IJ vein for the presence of the guidewire, as well as scanning the carotid artery for its absence, to reduce the likelihood of a devastating arterial injury. 

### Disclosures

The authors have no conflict of interest or relevant funding disclosures.
